# Geometrical versus Random β-TCP Scaffolds: Exploring the Effects on Schwann Cell Growth and Behavior

**DOI:** 10.1371/journal.pone.0139820

**Published:** 2015-10-07

**Authors:** Lauren Sweet, Yunqing Kang, Christopher Czisch, Lukasz Witek, Yang Shi, Jim Smay, Giles W. Plant, Yunzhi Yang

**Affiliations:** 1 Department of Orthopaedic Surgery, Stanford University, Stanford, California, United States of America; 2 Department of Biology, Stanford University, Stanford, California, United States of America; 3 Department of Ocean and Mechanical Engineering, Florida Atlantic University, Boca Raton, Florida, United States of America; 4 Department of Neurosurgery, Stanford University, Stanford, California, United States of America; 5 School of Chemical Engineering, Oklahoma State University, Stillwater, Oklahoma, United States of America; 6 Department of Materials Science and Engineering, Stanford University, Stanford, California, United States of America; 7 Department of Bioengineering, Stanford University, Stanford, California, United States of America; University of Texas at San Antonio, UNITED STATES

## Abstract

Numerous studies have demonstrated that Schwann cells (SCs) play a role in nerve regeneration; however, their role in innervating a bioceramic scaffold for potential application in bone regeneration is still unknown. Here we report the cell growth and functional behavior of SCs on β-tricalcium phosphate (β-TCP) scaffolds arranged in 3D printed-lattice (P-β-TCP) and randomly-porous, template-casted (N-β-TCP) structures. Our results indicate that SCs proliferated well and expressed the phenotypic markers p75^LNGFR^ and the S100-β subunit of SCs as well as displayed growth morphology on both scaffolds, but SCs showed spindle-shaped morphology with a significant degree of SCs alignment on the P-β-TCP scaffolds, seen to a lesser degree in the N-β-TCP scaffold. The gene expressions of nerve growth factor (β-ngf), neutrophin–3 (nt–3), platelet-derived growth factor (pdgf-bb), and vascular endothelial growth factor (vegf-a) were higher at day 7 than at day 14. While no significant differences in protein secretion were measured between these last two time points, the scaffolds promoted the protein secretion at day 3 compared to that on the cell culture plates. These results together imply that the β-TCP scaffolds can support SC cell growth and that the 3D-printed scaffold appeared to significantly promote the alignment of SCs along the struts. Further studies are needed to investigate the early and late stage relationship between gene expression and protein secretion of SCs on the scaffolds with refined characteristics, thus better exploring the potential of SCs to support vascularization and innervation in synthetic bone grafts.

## Introduction

The key challenge of bone tissue regeneration using ceramic scaffolds is still insufficient vascularization [1,2]. While many strategies are being developed, one avenue that has not been extensively explored in bone graft engineering is the incorporation of nervous system glia and their potential role in bone tissue regeneration. Studies have shown that, throughout the body, nerves and blood vessels run in parallel. Even in cases where nerves have been intentionally made to misalign, blood vessels will still follow the defective nerve pathway [3]. This suggests a significant role of the nervous system in angiogenesis [4–6], which is pertinent due to angiogenesis’s key role in osteogenesis [7]. Additionally, nerves run throughout all three layers of the bone (periosteum, cortical bone and trabecular bone) and studies have shown that they serve a significant function in their coordination with bone metabolite cells—the osteoblasts and osteoclasts [8,9]. Of the cell types specific to the nervous system, Schwann cells (SCs) have great potential to assist in the creation of an integrated, multi-system bone graft. SCs are already widely known to provide not only innervation to grafts through their role in peripheral nerve repair [10–12], but also significant angiogenic inducing potential through growth factor secretion [13].

SCs are the myelinating cells of the peripheral nervous system (PNS); they wrap around axons to improve signal conduction as well as assist in neural regeneration after injury in the PNS [14,15]. Additionally, SCs have been shown to play a pivotal role in arteriogenesis in the skin—without them, proper artery formation fails to occur [3,16]. SCs can also produce the extracellular matrix needed for effective neurite ingrowth [17]. Moreover, they secrete a wide variety of growth factors, including but not limited to brain-derived neurotrophic growth factor (BDNF), nerve growth factor (NGF), neutrophin–3 (NT–3), hepatocyte growth factor (HGF), platelet-derived growth factor (PDGF), vascular endothelial growth factor (VEGF), insulin-like growth factor 1 and 2 (IGF–1, IGF–2) and glial cell line-derived neurotrophic factor (GDNF) [18–24]. All these factors make SCs promising candidates in a graft that integrates both nerves and vessels.

It is most common for SCs to be grown on pliable substrates like hydrogels [25]. However, utilizing the favorable functionality of SCs in a bone graft would require these cells to be able to function in a rigid environment as well as a pliable one because optimal use of the SCs behaviors will occur if they can interact with cells in all areas of a graft. Although SCs have been grown on a variety of semi-rigid and rigid materials such as poly(lactic-co-glycolic) acid (PLGA), polycaprolactone (PCL), polylactic acid (PLA), collagen and nano-fibers [25–30], their viability and behavior on a β-tricalcium phosphate (β-TCP) scaffold have yet to be evaluated. β-TCP synthetic grafts have been widely used in clinical settings for bone repair and regeneration due to a high degree of bio-compatibility, bioactivity, relative strength and mimicry of natural bone minerals [31–33]. Thus, it is important to understand the SC reaction to such a material if further integration of this cell type is to be applied to future bone tissue engineering.

Additionally, in regards to SC behavior, studies have shown that directed fibers or patterned scaffolds facilitate the functional performance of SCs [34]. This patterning can induce multiple SC behaviors including cellular alignment, migration and the SCs’ effectiveness at directing and inducing axonal ingrowth [35–37]. Therefore, the first goal of this study is to evaluate the feasibility of growing SCs on a favorably osteoconductive β-TCP ceramic scaffold and characterize the SCs’ behavior on such a bioceramic material. The second goal of this study is to investigate the effect of strut arrangement in porous scaffolds on SCs’ alignment. Two different geometries were chosen to determine what type of scaffold would be able to induce the best SC alignment. Since end-to-end cell alignment is an important part of SC functionality in the regrowth of injured peripheral nerves [34], the ability of the scaffold to guide SC orientation is of relevance.

We hypothesized that biodegradable, porous ceramic β-TCP scaffolds can support SCs’ growth and related function. Furthermore, we examined the effect of scaffold geometry on SCs behavior. To this end, we fabricated a patterned β-TCP scaffold (patterned β-TCP, P-β-TCP) using a 3D printing method and a porous β-TCP scaffold using a template-casting method (non-patterned β-TCP, N-β-TCP). We characterized the cell attachment, proliferation, migration, protein secretion and alignment on these scaffolds to assess whether the β-TCP material caused any significant deviations in normal SC behavior.

## Materials and Methods

### Ethics Statement

The protocol of isolating rat Schwann cells from adult Fischer rats (F344) sciatic nerves was approved by the Institutional Animal Care and Use Committees (IACUC) of Stanford University, and the administrative panel on laboratory animal care (APLAC) of Stanford University also approved this work.

### Preparation of β-TCP scaffolds

The porous N-β-TCP scaffolds were fabricated using the template-casting technique previously described [32,38]. In brief, a ceramic slurry consisting of 12 grams β-TCP powder (Fisher Chemical), 1 mL surfactant (Surfonal^®^), 1mL dispersant (Darvan^®^ C) (R.T. Vanderbilt), and 2.4 grams carboxymethyl cellulose powder (Fisher) were combined with water under heat and constant mixing. After the mass ratio of water to added solid powders reached 2.5, the β-TCP slurry was cooled at 4°C. The slurry was then cast into a customized mold formed by packed paraffin beads that had been subjected to slight melting via the application of low heat. The slurry was cast using a vacuum before undergoing a graded dehydration in heated ethanol for two hours per solution (70%, 90%, 95% ethanol solutions). During this process, the paraffin melted out and the remaining ceramic green bodies were dried for two hours before being sintered in a high temperature kiln at 1250°C for 3 hours.

The patterned P-β-TCP scaffolds were fabricated via a 3D printing method utilizing a direct write printer (Aerotech Inc., Pittsburgh, PA) to extrude the colloidal ink through a nozzle. The first step in preparing the P-β-TCP scaffolds was to calcine and mill the raw β-TCP (Sigma-Aldrich, St. Louis, MO) powder. The β-TCP colloid gel was prepared with a volume fraction of 47%, by mixing the correct amount of ceramic powder and ammonium polyacrylate (Darvan 821A, RT Vanderbilt, Norwalk, CT), 16 mg/gram ceramic, solution to allow for the dispersion of particles into the DI-water. Upon verifying all the calculations the first step was to add 20 grams of milling media into a mixing cup (THINKY, Tokyo, Japan) followed by the predetermined amount of ceramic powder, DI-H_2_O, and dispersant, subsequently mixing in a planetary mixer (Thinky AR–250, THINKY, Tokyo, Japan) for 3 minutes at 2000 rpm. After mixing the slurry was allowed to cool for 5 minutes in a water bath, followed by the addition of a thickening agent, hydroxypropylmethylcellulose (Methocel F4M, Dow Chemical Company, Midland, MI). The hydroxypropylmethylcellulose was in the form of a 5% weight solution, and added to the slurry at the proportion of 7.5 mg per milliliter of ceramic, spun for 1 minute and allowed to cool in water bath. The last step in preparing the slurry was to gel the suspension by adding a 10% weight solution of polyethyleneimine (9002-98-6, Sigma-Aldrich, St. Louis, MO) at a ratio of ~175 mg per 30 mL of colloid gel prepared followed by spinning for 1 minute. The colloidal gel was then transferred into a 3 mL syringe (EFD Inc., Nordson, Westlake, OJ), which was attached to the gantry of the microprinter that extruded the gel into a predesigned pattern designed using a custom computer-aided design (CAD) (RoboCAD 4.1, 3D Inks, Tulsa, OK) software. The computer used a controlled extrusion system, depositing ‘*printing*’ these scaffolds in a layer-by-layer fashion at a velocity of 8 μm/s into a paraffin oil to prevent drying of the scaffold during the extrusion process. The 3D fabricated green body underwent a similar sintering procedure at 1250°C for 3 hours.

### Measuring scaffold porosity and morphology

Porosity of the scaffolds was measured using the method previously described [39]. Briefly, scaffolds of a known weight (W_s_) were immersed in water of a known volume. The change in volume (ΔV) upon immersion of the scaffold in water was then recorded following evacuation of the air bubbles using vacuum pressure. Using the theoretical density of β-TCP (*ρ*_t_) and the measured bulk density (*ρ*_b_ = W_s_/ΔV) the porosity was calculated according to %porosity = (1-*ρ*_b_/*ρ*_t_) ×100 as described in [40]. Data was collected from 5 scaffolds per group. A scanning electron microscope (SEM) (FEI, USA) using the 15kV voltage setting was used to observe the pore morphology and measure the strut size of the scaffolds.

### Scaffold Degradation

Scaffold degradation was conducted by measuring the Ca^2+^ concentration in the surround medium. Scaffolds were placed in Dulbeco’s Modified Eagle Medium (DMEM) (Life Technologies^™^) without Fetal Bovine Serum (FBS) and incubated at 37°C. The released medium was collected at the designated time points and the same amount fresh medium was added to keep the volume constant. Five scaffolds were sampled in each group. Ca^2+^ measurements were taken using a Calcium Colormetric Assay Kit (Sigma Aldrich^®^) according to the provided protocol. Base Ca^2+^ concentration from the DMEM were also taken into account and measured (data not shown). Samples were then read on an Infinite^®^ F50 chemilluminscence plate reader (TECAN) and concentration analyzed according to the suggested procedure.

### Cell culture

Adult rat SCs were harvested from the sciatic nerve and purified according to the established protocol in our previous studies [41,42]. In this study, purified SCs were cultured in Dulbeco’s Modified Eagle Medium (DMEM) (Life Technologies^™^) supplemented with 10% heat inactivated FBS (Life Technologies^™^), 1% Glutamax (Life Technologies™), 1 μL/mL Gentamicin (Life Technologies^™^), and mitogens including 2 μL/mL Bovine Pituitary Extract (Life Technologies^™^) and 13.3 μL/mL of Forskolin (diluted to 6.25 mg/mL in DMSO) (Sigma Aldrich^®^) at 37°C and 5% CO_2_ [41]. SCs were cultured until 85–90% confluence and then passaged using 0.05% Trypsin EDTA. Culture surfaces were incubated overnight in poly-L-lysine (PLL) (1 mg/mL in water) (Sigma Aldrich^®^), which helps the cells adhere and migrate. It has been found that without PLL, approximately one-half of SCs will detach from the culture surface during growth [43]. 1x10^6^ cells in 1 mL medium were directly loaded onto the scaffolds and then submerged in culture medium/cell solution. Every 15 minutes for the first hour, the submerged scaffolds were agitated via directly pipetting the surrounding cell-containing supernatant back onto the scaffold. The scaffolds were flipped after the first 15 minutes. All cells used were at passage 6; cell medium was changed every 3 days.

### Live/Dead staining

A live/dead fluorescent solution of calcein-AM and Ethidium homodimer–1 was prepared according to the manufacturers instructed dilutions (Invitrogen™). Scaffolds were then submerged in the solution and incubated at 37°C for 15 minutes before being washed in phosphate buffered saline (PBS) and visualized using a fluorescent microscope (AxioObserver Z.1, Zeiss).

### SEM visualization

The SC-seeded scaffolds were fixed in 2.5% glutaraldehyde diluted in PBS. After washing with PBS, the samples underwent a graded dehydration in 70%, 80%, 95% and 100% ethanol for 20 minutes per solution. Samples were dried, sputter-coated in gold and examined with SEM.

### Immunofluorescent staining

Samples were collected at days 7 and 14, washed and fixed in 4% paraformaldehyde for 15 minutes at room temperature. Following fixation, cells were treated with 0.5% Triton X100 and then blocked with 5% BSA/PBS, washed, stained with anti-p75^LNGFR^ rabbit antibodies (1:200 dilution) (Promega) in 1% BSA/PBS buffer and incubated overnight at 4°C. The samples were then stained with green goat anti-rabbit Alexa-Fluor 488 secondary antibodies (Invitrogen™). The samples were then blocked with 5% goat serum/PBS buffer. Anti-S100-β mouse antibodies (1:1000 dilution) (Sigma Aldrich^®^) in 1% BSA/PBS buffer were then added and incubated overnight at 4°C. After a thorough washing, red goat anti-mouse antibodies Alexa Fluor 594 (Invitrogen^™^) were added (1:1000 dilution) and incubated for 1 hour at room temperature. Samples were then subjected to a DAPI (5 μg/mL) stain. Cells were then visualized under a fluorescent microscope (AxioObserver Z.1, Zeiss). The percent of the image positive for a certain antibody color was calculated using the ImageJ (NIH) plug-in. Two samples per group from each time point were used. “Color Pixel Counter” (Ben Pichette), and differences in color percentages between the S100 fluorescence (red pixels) and p75 fluorescence (green pixels) in the experimental groups were compared to this same difference in the control group.

### dsDNA assay

Cells/scaffolds were collected at 3, 7, and 14 days; they were washed and frozen at -80°C. They were then subjected to 3 freeze/thaw cycles at -80°C /37°C. DNA was extracted using 0.5% Triton X–100 and dsDNA concentration was detected by Picogreen assay (Molecular Probe, Invitrogen™) according to the manufacturer’s specified concentrations. The mixture was incubated for 5 minutes before being read on an Infinite^®^ F50 chemilluminscence plate reader (TECAN) with an ex/em of 480/520 nm.

### Real-time PCR

Total RNA was extracted using QIAshredder (Qiagen^®^) and RNeasy Mini Kit (Qiagen^®^) following the manufacturer’s instructions. The RNA concentrations were then measured using an Eppendorf^®^ Biophotometer. An iScript cDNA synthesis kit (Bio-Rad^®^) was used to reverse-transcribe RNA of the samples into cDNA according to the manufacturer’s protocols. Then using the cDNA product template, specific primers, and iQSYBR Green supermix (Bio-RAD), a real-time quantitative PCR reaction was performed on an Applied Biosystems^®^ (ABI) 7900HT Fast Real-Time PCR System (ABI). The total reaction volume was 10 μl according to the manufacturer’s directions. Primers were sourced from Elim Biopharmaceuticals, Inc.. Gapdh was used as a housekeeping gene. (pdgf-bb forward: AGACGAAGATGGGGCTGAGCTG, pdgf-bb reverse: CACTGCACATTGCGGTTATTGC [18], nt–3 forward: GGTTGCAGGGGGATTGAT, nt–3 reverse: TATTCGTATCCAGCGCCA [18], ngf-β forward: CTTCAACAGGACTCACAGGAGCAA, β-ngf reverse: TGGTCTTATCTCCAACCCACACAC [19], vegf-a forward: CCAAAGCCAGCACATAGGAGAGAT, vegf-a reverse: CCCTTTCCCTTTCCTCGAACTGAT [19], gapdh forward: TCAACGGCACAGTCAAGG, gapdh reverse: TGAGCCTTCCACGATG [44]). The relative gene expression levels of the various genes (nt–3, β-ngf, vegf-a, pdgf-bb) were normalized to the endogenous house-keeping gene gapdh. Gene expression of the tissue culture plates at each individual time point was designated as the control group. Their relative levels of expression were then calculated using the 2^-ΔΔCt^ method and equation where ΔΔCt is derived via the equation (Ct,_target_-Ct, _control_)_target gene_-(Ct,_target_-Ct,_control_)_GAPDH_ as described by Livak and Schmittgen [45].

### Cell alignment

Images of DAPI staining taken from 4 random microscope fields were chosen from among two separate samples for each group. Each DAPI image was then analyzed using the angle tool in ImageJ [46]. Upwards of 375 cells were measured for each group per time point. For each cellular nucleus, an angle was drawn between the major axis of the elliptical nucleus and the corresponding line drawn through the predominating strut direction. The corresponding angle off the strut was then recorded.

### ELISA test

ELISA tests were performed on the supernatant media collected at days 3, 7, and 14. Three samples from each group for each time point were used. Tests for rat VEGF-A and rat β-NGF were run following the manufacturer’s instructions (Sigma-Aldrich^®^). Briefly, media was added to wells containing plate-bound antibodies specific to the protein. This was incubated overnight and after washing, biotinylated anti-growth factor antibody was added followed by subsequent incubations, washings and addition of HRP-conjugated streptavidin, and HRP substrate. The amount of VEGF-A and β-NGF were calculated by comparing the standard curves of the known VEGF-A and β-NGF sample according to the manufacturer’s instructions, and the secreted concentration per cell was normalized using dsDNA content

### Statistical analysis

In this study, all the experiments were performed in triplicates unless otherwise noted, and two and three way ANOVA tests followed by a Holm-Sidak comparison test was used to analyze the statistical significance. If the *p*-values were less than 0.05, the difference was considered to be significant.

## Results

### β-TCP morphologies

**Fig 1:**
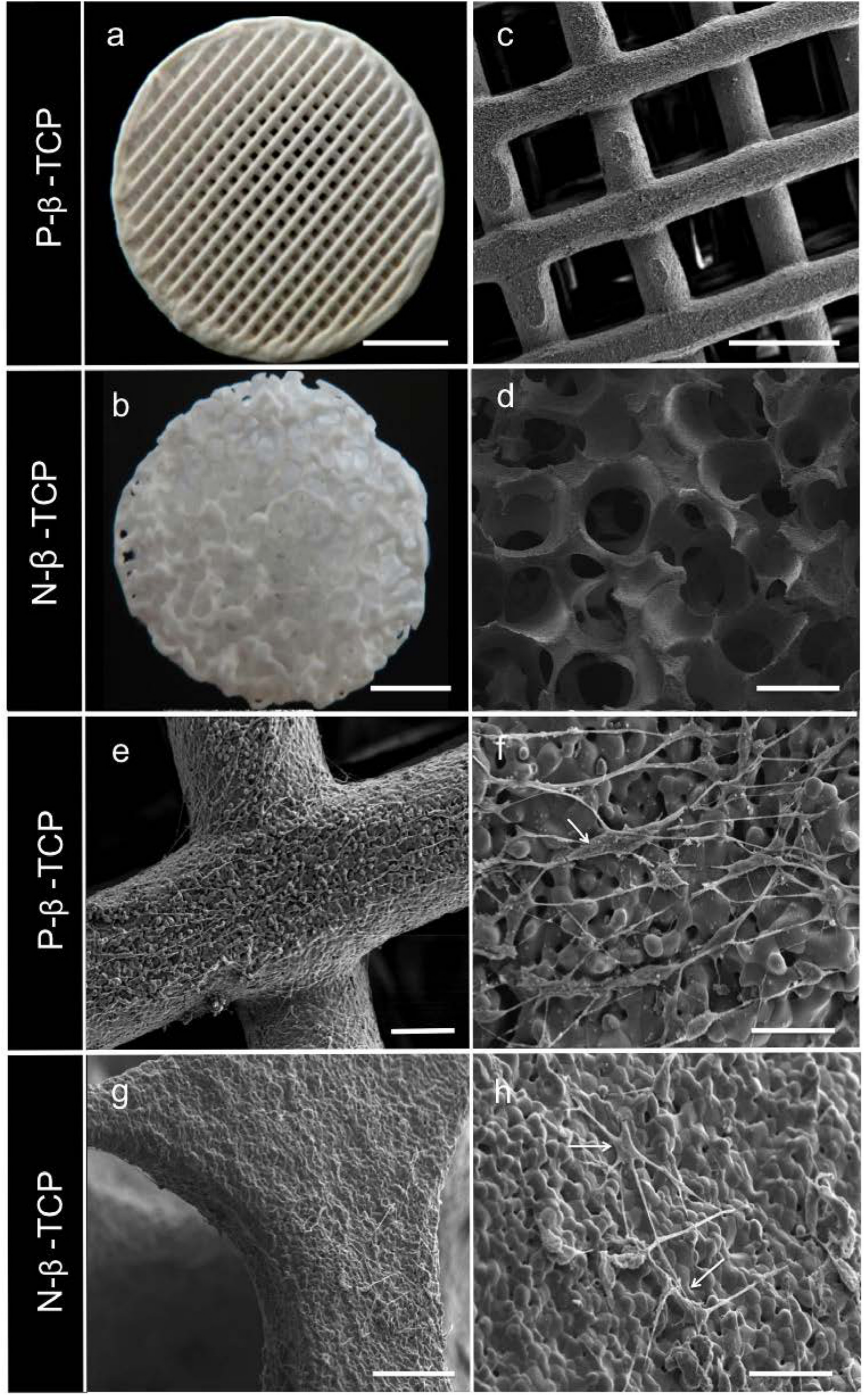
SEM and Microscopy Imaging. Micro and SEM images of the printed and template casted scaffolds at low (a, b) and high magnification (c, d) and at day 5 (e-h). Day 5 images show visual alignment of SCs on the printed scaffold. Arrows indicate SCs on scaffolds. Scale bar on a, b = 2mm; on c, d = 500μm; on e, g = 100μm; on f, h = 25μm.

**Fig 1** shows the differences in both scaffold surfaces under SEM. The diameter of the N-β-TCP and P-β-TCP scaffold is around 8 mm. Pores of the template-casted scaffold range from 350–500 μm with a strut width measuring 110 ± 94 μm, which is similar to the previously measured value of 140 ± 84 μm [32]. The 3D-printed scaffold had rectangular pores of 400 μm and a strut width of about 230 ± 15 μm. The structural configurations of the β-TCP scaffolds can be seen under higher magnification (**Fig 1C and 1D**). Microscopic visualization of the surface of both printed and casted scaffolds showed that, while both constructs had similarly sized micrograins, the surfaces of the P-β-TCP structures appeared marginally smoother and more regular than those of the casted N-β-TCP scaffolds (**Fig 1F and 1H**). Porosity of the N-β-TCP and P-β-TCP scaffolds was measured at 79.2±2.3% and 42.3±6.7%, respectively.

### Cell morphologies on β-TCP scaffolds

SEM images in **Fig 1** show that cells attached and grew on the scaffolds. Samples of both N-β-TCP and P-β-TCP scaffolds were taken at day 5. The bipolar nature of the SC bodies and their attachments to the surface of the material are visible at higher magnification. Results show that SCs demonstrated apparent aligned direction on the struts of P-β-TCP scaffolds and spindle-shaped morphology (**Fig 1E and 1F**), whilst on the N-β-TCP scaffold, SCs grew without any visually obvious alignment and showed polygon morphology (**Fig 1G and 1H**).

### Live/Dead cytotoxicity assay

**Fig 2:**
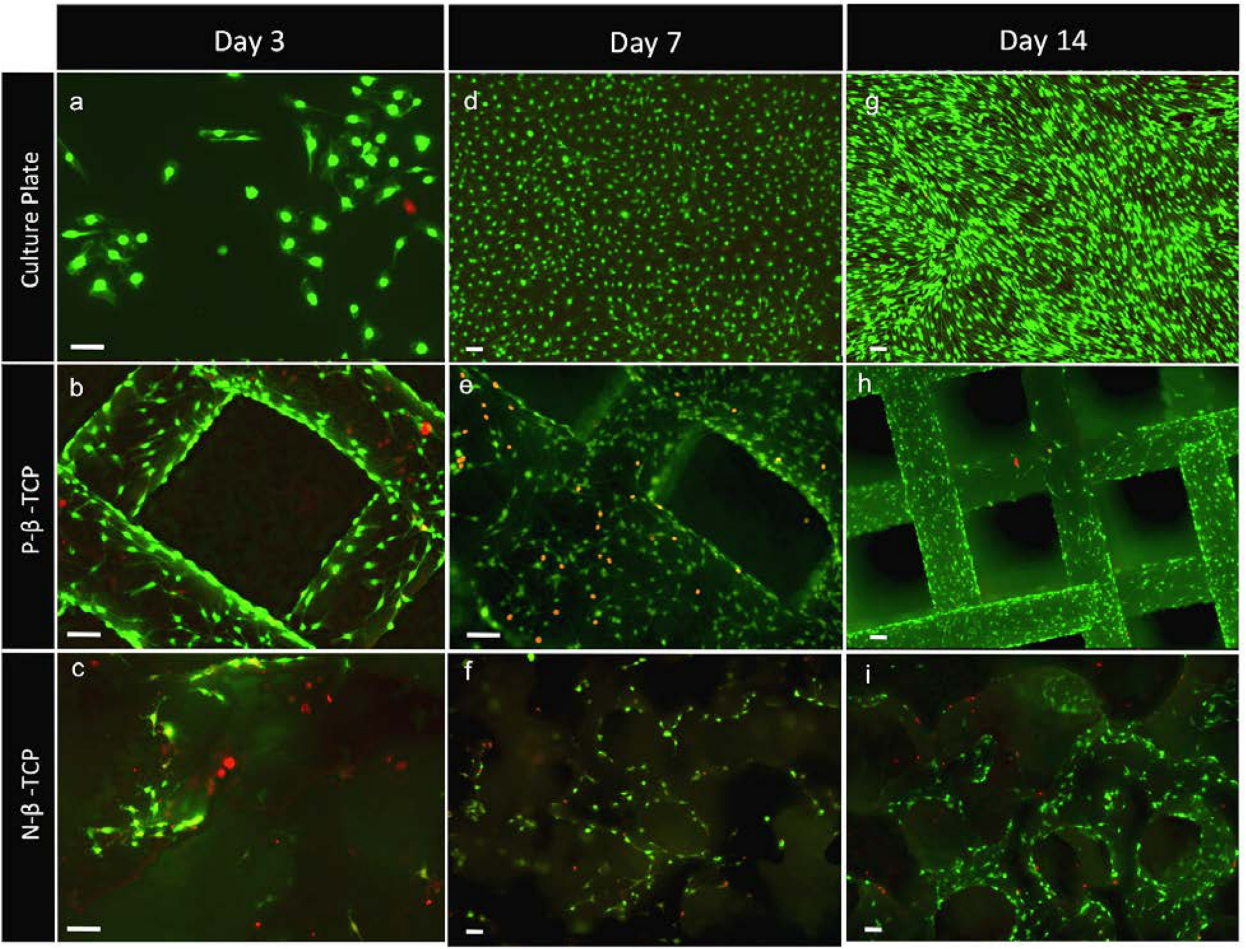
Live/ Dead cytotoxicity viability assay at days 3 (a, b, c), 7 (d, e, f), and 14 (g, h, i). Staining also suggested directed growth of the cells on the 3D-printed scaffold in parallel with the struts. Staining highlights pattern of clustered growth on the scaffolds (e, f, h, i). Scale bar = 100μm.

To further test the toxicity of β-TCP material, we used a dense, solid disk of β-TCP as a control. All tests for this group produced results similar to those of the constructs, and in some cases produced increased responses. In short, the dense material had no apparent effect on the cells’ growth or function. Toxicity data for the β-TCP disk can be found in **Figure A and B in S1 File**. A live/dead cytotoxic assay was conducted at days 3, 7, and 14. Results in **Fig 2** show that SCs grew and proliferated well on the scaffolds with time and the majority of cells remained viable at all time points tested, although the fraction of dead cells appeared to be greater on the P-β-TCP and N-β-TCP scaffolds than that on tissue culture plates. Results further show that P-β-TCP appeared to be more confluent at days 3, 7 and 14 compared to the N-β-TCP scaffolds. Additionally, cell density appeared less consistent on porous N-β-TCP than that on P-β-TCP scaffolds, with cells tending to settle and grow in clusters on the N-β-TCP scaffolds while exhibiting more uniform migration and coverage on the P-β-TCP constructs.

### Immunofluorescent staining

**Fig 3:**
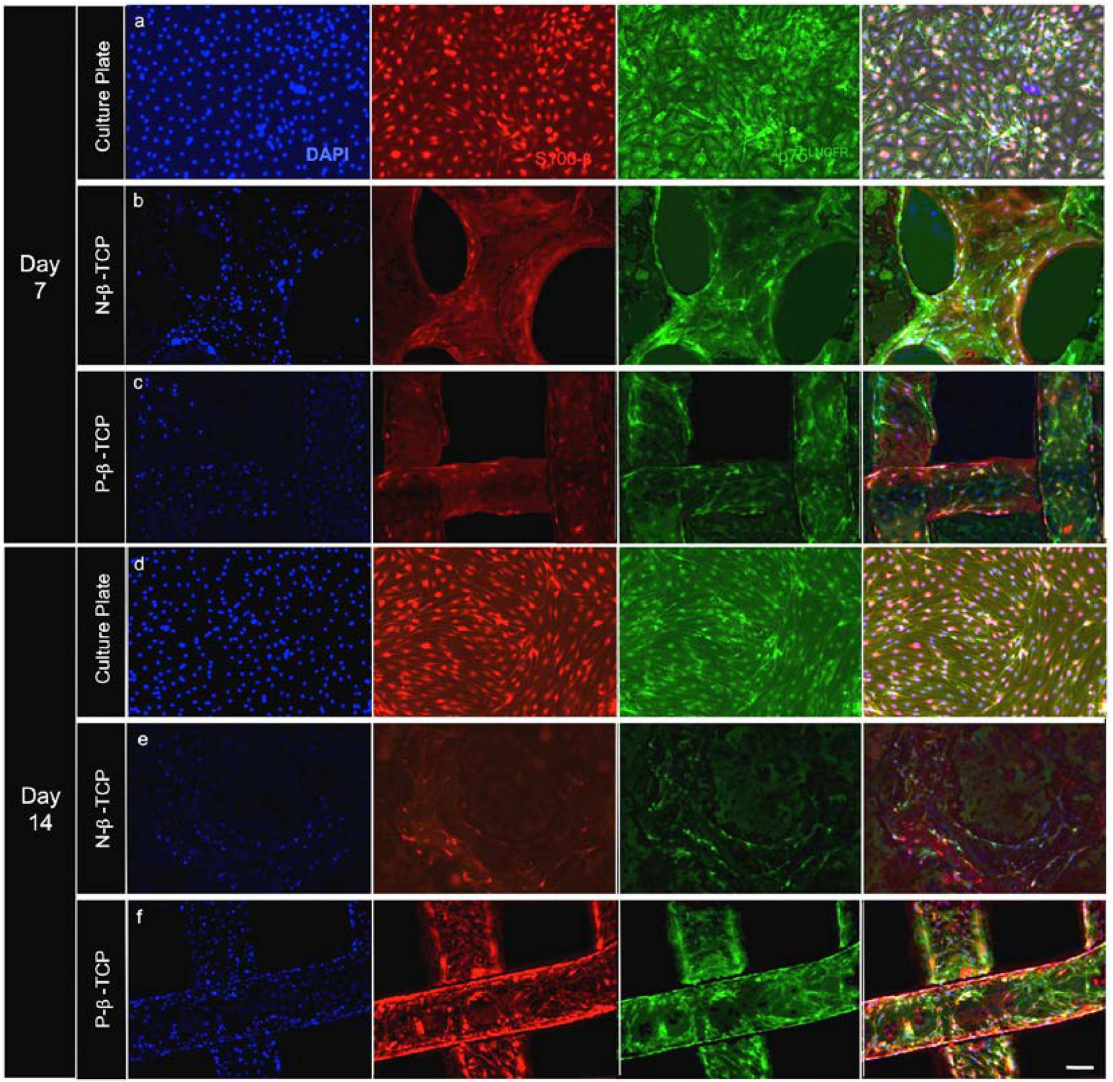
Immunostaining for p75^LNGFR^(green) and S100-β (red) for days 7 (a-c) and 14 (d-e). Panels are divided horizontally by group and vertically by stain. The final column is a co-stain of DAPI, S100-β, and p75^LNGFR^. Majority of cells stain positive for both SC markers suggesting the scaffolds do not significantly influence cell character. Scale bar = 100μm.

A double staining for proteins S100-β and p75^LNGFR^ were performed on all samples at 7 and 14 days (**Fig 3**). The S100-β subunit is a cytosolic calcium-binding protein that plays an important role in growth, cell signaling, movement and metabolism [47]. P75^LNGFR^ is a low-affinity neurotrophin receptor on the surface membrane of SCs that can bind various growth factors such as β-NGF or NT–3 [48]. As seen in **Fig 3**, all groups had obvious staining for both these proteins. Most cells showed positive expression for both proteins, which suggest that the β-TCP does not change normal SC identity. No significant difference in staining percentages was seen between the experimental scaffolds when compared to the staining percentages seen in the control groups. On average, the difference in pixel percentages between S100-β (red pixels) and p75^LNGFR^ (green pixels) staining area was 4.47% for all groups. This is not significant as it is less than 5% of the total area and well within the bounds of experimental image error.

### Cell alignment

**Fig 4:**
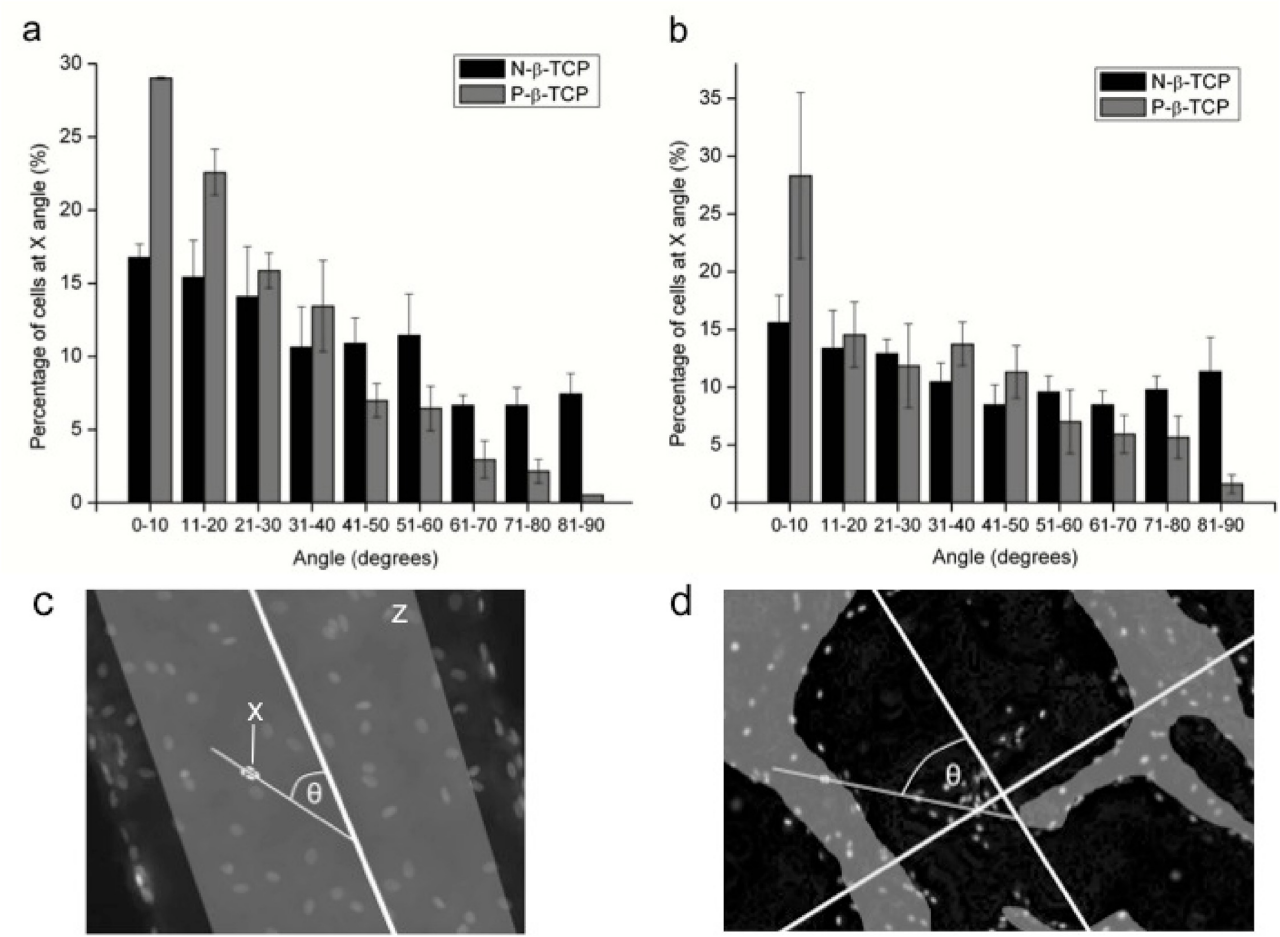
Cell Alignment. Results show the percentage of cells in a given random viewing field within a certain degree of parallel with the major scaffold struts. Data is from DAPI images taken at 7 (a) and 14 (b) days from 4 different fields per time point. Fields analyzed were different than those in Fig 3. Schematic (c-d) shows the method of measuring the angle between the cell axis in relation to strut. X marks the location of the cell being measured. A line was draw through the major axis of the cell’s nucleus and the angle this line formed with the indicated strut was recorded. The shaded region denoted by Z indicates the top facing regions of the strut from which nuclei were measured.

Cell alignment data showed a correlation between cell orientation and scaffold type (**Fig 4**). Cell directionality was derived from the angle of the cell nuclei measured in relation to the strut. It was found that the majority of SCs on the P-β-TCP scaffold grew at an angle between 0 and 30 degrees off-parallel from the strut. This parallelism with the P-β-TCP scaffold’s internal configuration was seen to have the tightest connection at day 7, where approximately 51% of cells were within 20 degrees of parallel with the strut (**Fig 4A**). While there was some observed alignment on the struts of the casted N-β-TCP scaffold, the correlation is much less defined than in the P-β-TCP groups—albeit the N-β-TCP construct data is still indicative of a material influence on the SCs. By day 14, the strong effect on directionality exhibited by both scaffolds, although most notably the P-β-TCP scaffold at day 7, appears to decrease slightly. However, the data is still suggestive of an induced directionality (**Fig 4B**).

### Cell proliferation and dsDNA

**Fig 5:**
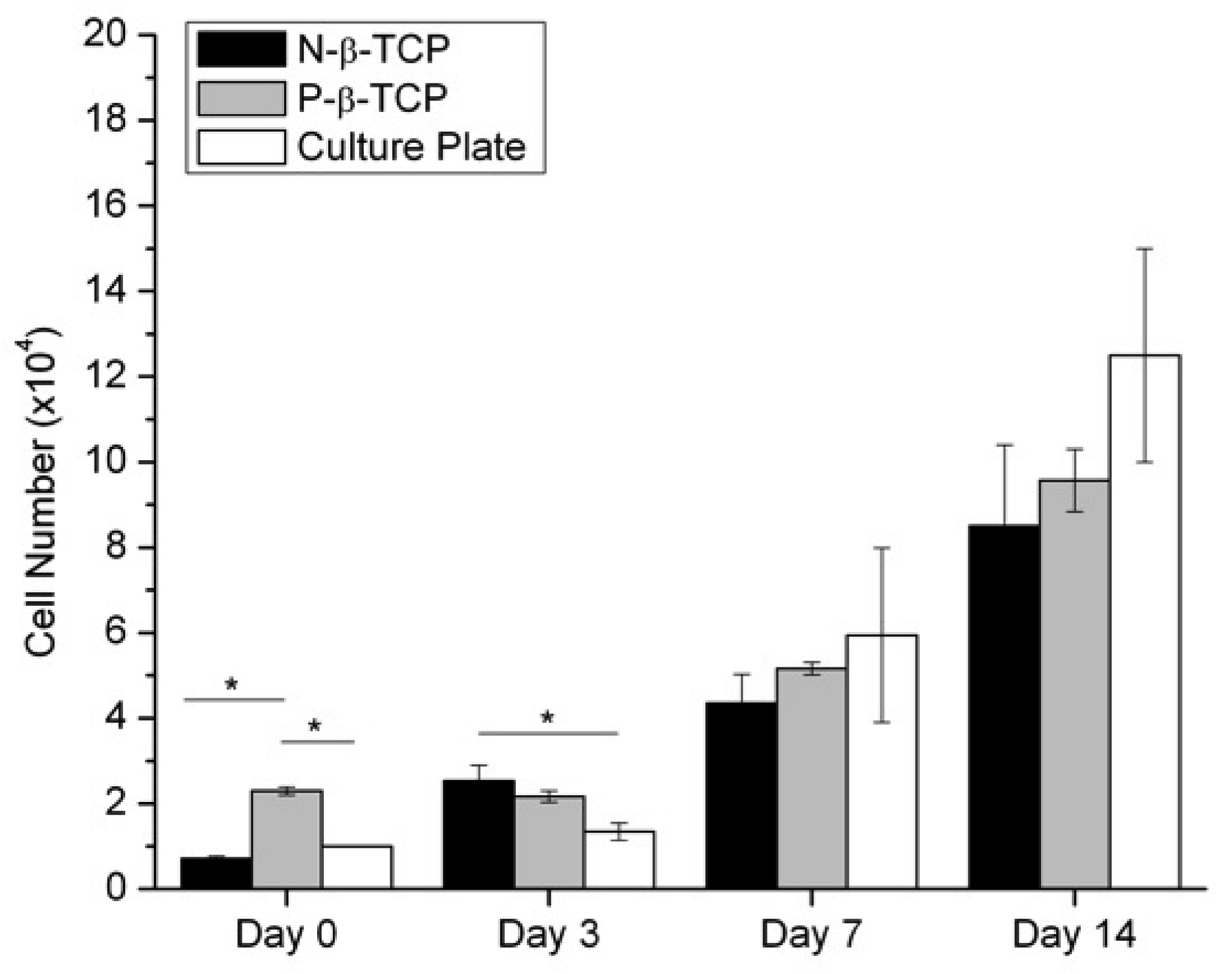
dsDNA Assay. Graph shows cell number at each time point. Starred bars designate significant differences between groups within a single time point (n = 3).

The dsDNA assay results indicated a steady trend of growth over the course of the 14 days’ experiment for all groups. There was no significant difference in dsDNA measurements between the groups after day 3 (**Fig 5**), although the number of cells initially loaded on the scaffolds varied significantly (7.1% attachment for N-β-TCP of 79.2±2.3% porosity versus 22.9% attachment for P-β-TCP of 42.3±6.7% porosity), thus demonstrating that β-TCP does not significantly impact the viability and support growth of SCs. This result is consistent with the observation of live/dead staining as shown in **Fig 2**.

### Gene expression analysis

**Fig 6:**
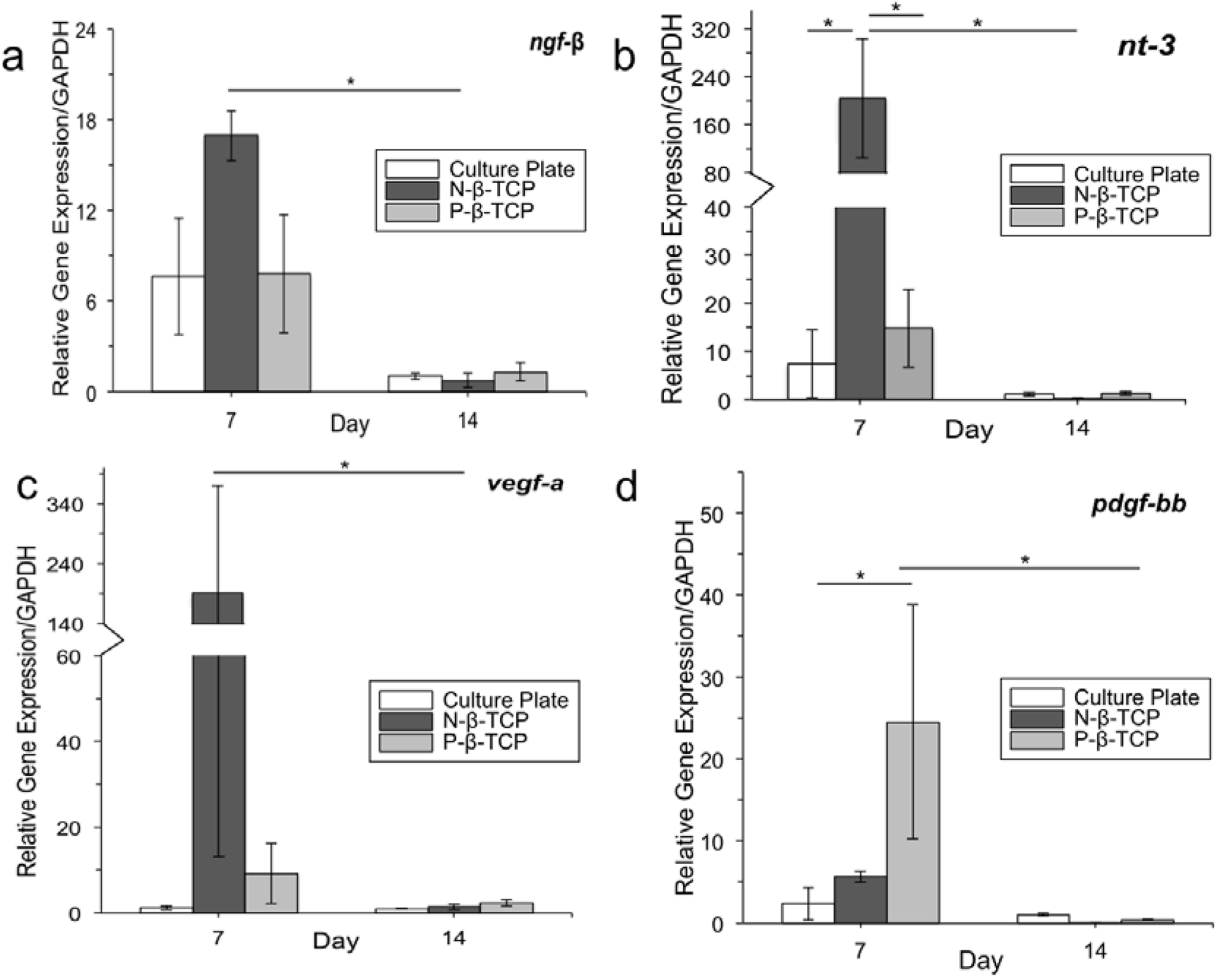
RT-PCR Assay. Four genes were analyzed: ngf-β (a), nt–3 (b), vegf-a (c), and pdgf-bb (d) with GAPDH used as a house-keeping gene. Data is shown as expression relative to GAPDH at 7 and 14 days. Starred bars specify significant differences between groups.

The gene expression of vegf-a, ngf-β, nt–3 and pdgf-bb was measured at day 7 and day 14. Results show that on the scaffold groups, the mRNA gene expression of the four genes at day 7 appears to be of a higher magnitude than that at day 14 (**Fig 6**). The gene expression of ngf-β on the N-β-TCP scaffolds at day 7 is significantly higher than that at day 14, but there is no difference between the three experimental groups at the two time points (**Fig 6A).** Additionally, the gene expression of nt–3 on the N-β-TCP group is significantly higher than those in the control and P-β-TCP groups at day 7, a difference which disappeared at day 14 (**Fig 6B**). There is no difference in the gene expression of vegf-a between the three groups at day 7 and 14, but its expression on the N-β-TCP scaffolds shows significant decreases at day 14 compared to that at day 7 (*p*<0.05) (**Fig 6C)**, Exceptionally, P-β-TCP scaffolds but not N-β-TCP scaffolds significantly promoted the expression of pdgf-bb gene at day 7 compared to the control group (*p*<0.05) (**Fig 6D)**.

### Protein concentration using ELISA assay

**Fig 7:**
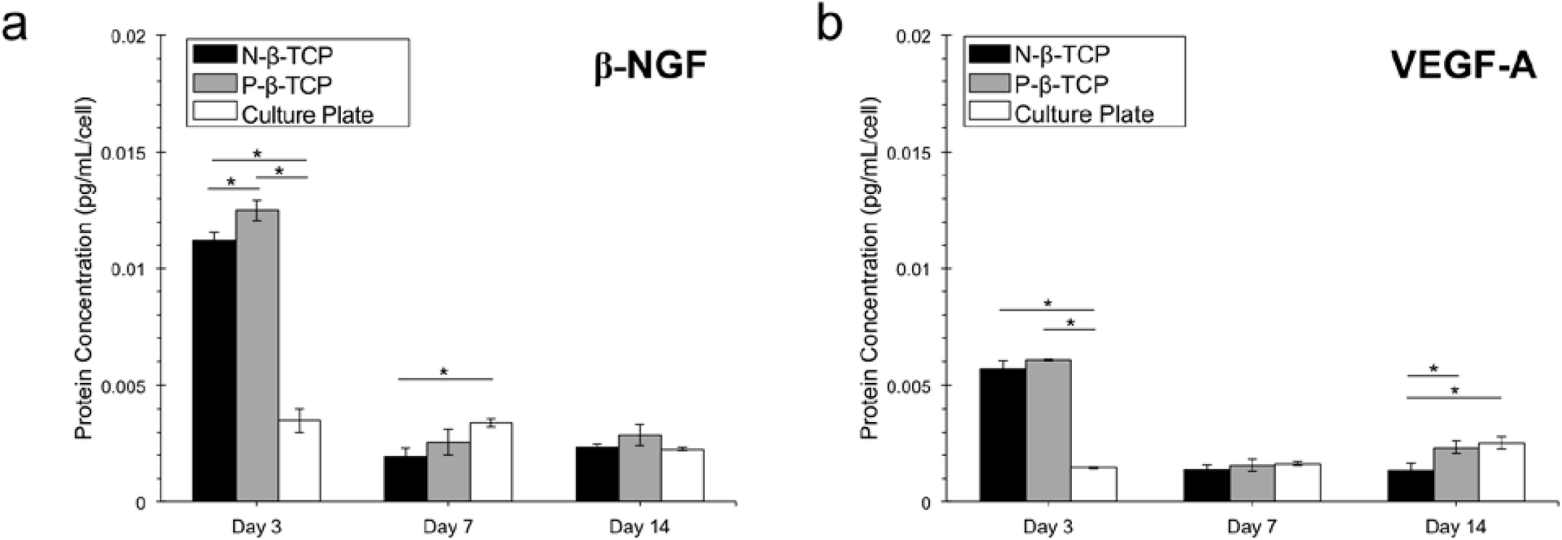
ELISA Assays for β-NGF (a) and VEGF-A (b). Protein concentration per cell in the growth media at days 3, 7, and 14, was normalized using the cell numbers from the dsDNA (Fig 5) results. Data collected from three different samples at each time point. Starred bars indicate significant difference between groups at each time point. Significant differences between time points not shown.

In this study, we chose two important proteins related to nerve growth (NGF), and vascular growth (VEGF-A). ELISA assay data shows that at day 3, β-NGF concentration in the P-β-TCP and N-β-TCP groups is significantly higher than that in the control group, while its concentration in the P-β-TCP group is higher than that in N-β-TCP groups (*p*<0.05) (**Fig 7A**). Whilst the concentration of β-NGF in the control group remained stable between day 3 to 14, the concentration in the scaffold groups experienced a slump. Although initial β-NGF concentration for the scaffolds groups began at the higher-than-control concentrations of about 0.013 pg/mL/cell, by day 7, these concentrations had significantly decreased by almost half to a concentration comparable to the culture plates. By day 14, all groups had similar protein outputs, with the culture plate actually having a significantly lower protein expression at day 14 than at day 7. The concentration of VEGF-A shows a similar pattern to the β-NGF data (**Fig 7B**). In both, the scaffolds experienced a slump in protein secretion followed by stabilization between days 7 and 14. At day 14, only the control group was secreting a greater amount of protein compared to its day 3 levels, whereas the differences in the N-β-TCP and P-β-TCP groups were significantly less than at day 3. The concentration of VEGF-A in P-β-TCP scaffolds is also significantly higher than that in N-β-TCP scaffolds at day 14 (*p<*0.05).

## Discussion

The results of this study show that β-TCP supports SC proliferation in addition to expression of the p75^LNGFR^ and S100-β phenotype, suggesting that the β-TCP material posed little-to-no toxicity to the SCs or change in characteristic phenotype. Another interesting observation of this study was the apparent alignment induced by the P-β-TCP scaffold, while the cells of the randomly-porous N-β-TCP scaffolds are less inclined to align. Under normal 2-D static conditions and high density, SCs use the cues from their neighboring cells to form parallel arrangements of cells that align their dendritic processes in the same direction [42]. The positive correlation between the lattice arrangement and cell alignment preference seen in the organized struts of the 3D porous scaffolds is probably a result of the combined influence of porosity, strut size, differences in surface topography and scaffold organization on cell adhesion, migration, and proliferation [49,50]. Meanwhile, to investigate the possible effect of the degradation rate of the two types of scaffolds, we studied the dissolution rate of P-β-TCP and N-β-TCP scaffolds at 37°C without cells. Results indicated that there is no significant difference between these two groups in degradation rate for the time period studied (Figure C in **S1 File**). This implies that the detected cell behavioral differences may not be affected by the degradation process of the β-TCP scaffolds but instead by the structure.

The cell alignment at the micron level seen in this study is especially promising for inducing nerve in-growth, in particular because inducing SC alignment has been a major focus for many nerve conduit engineers [29,34,51–53]. This is due to the natural mechanism of peripheral nerve repair wherein SCs in the injured area order themselves into strips known as Bands of Bünger, attracting and inducing local axons to grow down these aligned strands [12,54]. Thus, lattice struts with higher potential to direct SC growth hold great promise of attracting host axons into the construct. Already, the importance of this banding mimicry has been recognized as an important step for successful nerve conduits [28,55].

From the ELISA and RT-PCR results, we found that there is no clear evidence of a potential concentration dependent secretion pattern between expressed protein and the corresponding gene expression at 7 and 14 days. More specifically, it appears that N-β-TCP scaffolds slightly promoted the gene expression of the nerve-related gene β-ngf compared to P-β-TCP scaffolds but it did not promote the protein expression of NGF. While P-β-TCP scaffolds did not promote the gene expression of the vascular-related gene vegf-a compared to N-β-TCP scaffolds, it did seem to promote the protein expression of VEGF-A at day 14. This observational result may correlate with an autocrine-like behavior, which has been observed in previous SC studies [18], as well as with the fact that SCs both secrete and contain receptors for β-NGF [47,53,56]. However, early stage protein secretion at day 3 seemed not to correlate the following gene expression at day 7. Further studies are needed to investigate the early and late stage relationship between gene expression and protein secretion of SCs on the scaffolds with refined characteristics, thus better exploring the potential of SCs to support vascularization and innervation in synthetic bone grafts. Although the relationship between gene expression and protein secretion and the mechanism of autocrine-like correlation are still unknown here, the concentration of VEGF-A secreted in the P-β-TCP scaffolds may provide support for early angiogenesis, as the VEGF levels measured by the ELISA are similar to levels found to induce vessel growth in previous studies [57]. Further, since VEGF is considered one of the most powerful angiogenic proteins [58], this cell-regulated mechanism still has the potential to establish the basics of a microvasculature network without encountering the issues associated with excessive VEGF growth factor concentration, such as tumor formation or leaky vessels. Meanwhile, several studies have examined the positive role of VEGF in the survival of peripheral neurons and the stabilization of nerves [59–61]. SCs, being glial cells, also secrete the neuronal factors of β-NGF and NT–3. Thus, these secreted vascular growth factors could help to induce both angiogenesis and nerve survival in the graft. In an in vivo study done on rabbits, the presence on both endothelial cells and SCs allowed for increased sciatic nerve regeneration, showing increased vascularization as a possible cause [62]. Thus, SCs represent a cell with a possible dual-usage—one that could act supportively in vascularization and one that could directly stimulate re-innervation.

Of note, the variability in protein expression and secretion profiles seen in this study may be caused by differences in the surface areas of these groups. In addition, both of the scaffolds have 3D surfaces at the micro level, as opposed to the control groups (wells), which is a 2D environment. The difference in cell pattern growth is likely to have caused the differing protein outputs. Thus, increased interactions with neighboring cells potentially allow the SCs to engage in regulation mechanisms that help explain protein expression and secretion seen in the data.

The results of this study suggest that developing a better understanding of the benefits that SCs may be able to lend to the vascularization of bone grafts could lead to faster and more stable angiogenesis. Now that calcium phosphate (CaP) scaffolds appear to pose no significant hindrance to the growth of SCs, or their secretion of potent neural (β-NGF, NT–3) and angiogenic (VEGF-A, PDGF-BB) growth factors, more information is needed on the exact effect these cells might have on co-culture with endothelial cells (ECs). Specifically, further investigation into the proliferative and micro-vasculature-forming effects of SCs on ECs via growth factor secretion by SCs should be considered. Likewise, the effects on alignment and proliferation that ECs may have on SCs via growth factor interaction will also be a case for future study. Using the data on cell alignment discussed in this study, further experiments should be conducted as to whether there exists an optimal strut size or print arrangement to maximize or accelerate alignment of SCs, whether EC co-culture may enhance this alignment, and whether SCs could promote vascularization and network formation of ECs. Thus, this base-line, exploratory study on the growth of SCs on β-TCP scaffold has yielded novel and promising initial results for the future use of this cell-material pairing in creating a nervous/vasculature tissue-integrated bone graft.

## Conclusion

This study demonstrated that β-TCP scaffolds support the growth of SCs and normal nerve-related cell morphologies. While the printed scaffolds directed cell alignment more effectively, both 3-D culture systems induced distinct neural and angiogenic growth factor expression in comparison to the 2-D conditions. These initiatory results suggest that further exploration is needed into the possible angiogenic stabilizing properties of SCs to help create an innervated and vascularized graft.

## Supporting Information

S1 File Images of Live/Dead staining at days 3 (a, b), 7 (c, d), and 14 (e, f) of the β-TCP disk (b, d, f) and culture plate (a, c, e) show that no visible cytotoxicity is posed to the SCs by the dense β-TCP material, scale bar = 100μm (Figure A);Graph shows cell number at each time point for culture plate and β-TCP disk. There was no significant difference in cell number between the two groups at any time point, although day 14 was approaching a significant difference in cell population size (n = 3) (Figure B). Degradation rate of N-β-TCP and P-β-TCP scaffolds in culture medium DMEM without FBS (Figure C).
(DOCX)
