# Supplementary figures and images for: Geometrical versus Random β-TCP Scaffolds: Exploring the Effects on Schwann Cell Growth and Behavior
Source: PLoS One. 2015 Oct 7;10(10):e0139820. doi: 10.1371/journal.pone.0139820 (PMC4596809; doi:10.1371/journal.pone.0139820)

**Supporting Information**


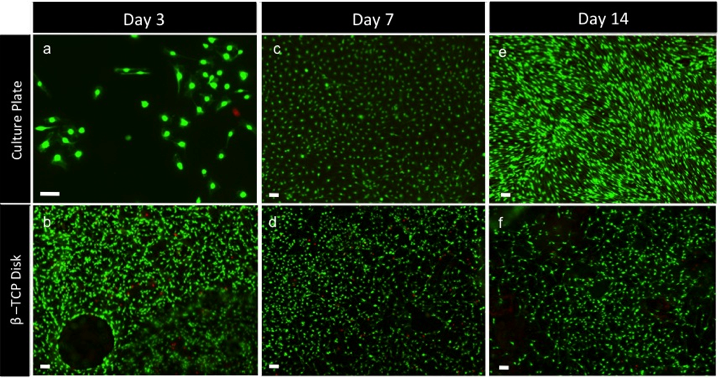


**Figure A**


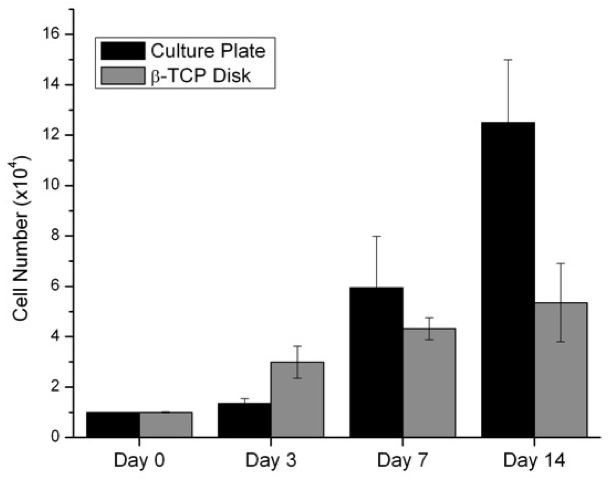


**Figure B**


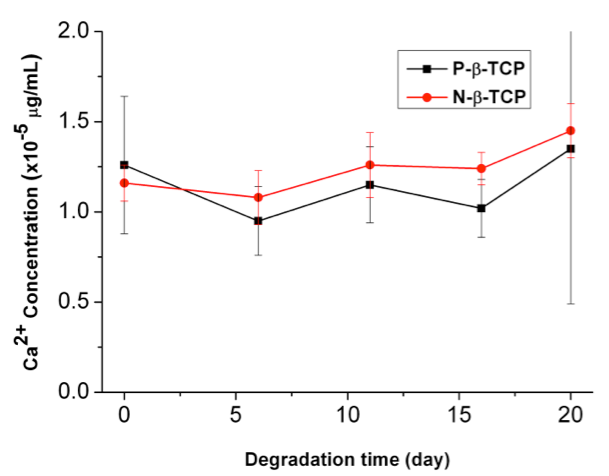


**Figure C**

Supplement: S1 File — There was no significant difference in cell number between the two groups at any time point, although day 14 was approaching a significant difference in cell population size (n = 3) (Figure B). Degradation rate of N-β-TCP and P-β-TCP scaffolds in culture medium DMEM without FBS (Figure C). (DOCX) [file pone.0139820.s001.docx]
